# The Relationship Between Blood Parameters and Gastrointestinal Bleeding in Atrial Fibrillation Patients Receiving Oral Anticoagulants

**DOI:** 10.3390/jcm14217642

**Published:** 2025-10-28

**Authors:** Hayrullah Yurdakul, Muhammet Cakas, Seda Elcim Yildirim, Tarik Yildirim, Suha Serin, Bahadir Caglar

**Affiliations:** 1 Nizip City Hospital, 27300 Gaziantep, Turkey; h.yurdakul@outlook.com; 2 Genc City Hospital, 12000 Bingol, Turkey; muhammetcakas@gmail.com; 3 Department of Cardiology, Faculty of Medicine, Balikesir University, 10900 Balikesir, Turkey; sedaelcimdurusoy@gmail.com (S.E.Y.); kdrtarik@gmail.com (T.Y.); 4 Department of Emergency Medicine, Faculty of Medicine, Balikesir University, 10900 Balikesir, Turkey; suhaserin@gmail.com

**Keywords:** atrial fibrillation, gastrointestinal hemorrhage, anticoagulants, biomarkers, emergency medicine

## Abstract

**Background/Objectives**: Atrial fibrillation (AF) is a prevalent cardiac arrhythmia associated with significant morbidity, including stroke, heart failure, and increased mortality, necessitating oral anticoagulant (OAC) therapy to reduce thromboembolic risk. However, OACs, including warfarin and non-vitamin K antagonist oral anticoagulants (NOACs), increase the risk of gastrointestinal (GI) bleeding, a serious complication requiring precise risk stratification in the emergency department (ED). **Methods**: This retrospective cohort study was conducted in the Emergency Department of Balikesir University Hospital in Turkey between 2019 and 2023 and evaluates systemic inflammatory markers as predictors of GI bleeding in AF patients receiving OACs. A total of 155 patients were divided into case (GI bleeding) and control (no GI bleeding) groups, comparing demographics, comorbidities, CHA_2_DS_2_-VASc and HAS-BLED scores, and inflammatory indices (uric acid/albumin ratio, neutrophil-to-lymphocyte ratio [NLR], platelet-to-lymphocyte ratio [PLR], systemic immune inflammation index [SII]). **Results**: For patients receiving NOACs, the case group exhibited significantly higher uric acid/albumin ratio, NLR, PLR, and SII (*p* < 0.05). For patients receiving warfarin, only the uric acid/albumin ratio was significantly elevated (*p* < 0.001). Hypolipidemia and elevated uric acid were associated with bleeding risk in patients receiving NOACs, while hypoalbuminemia and elevated urea predicted bleeding in patients receiving warfarin. HAS-BLED scores were significantly higher in bleeding groups, unlike CHA_2_DS_2_-VASc scores. **Conclusions**: These findings suggest that inflammatory indices, particularly in patients taking NOACs, are associated with GI bleeding risk stratification. Integrating these biomarkers into clinical practice could optimize personalized anticoagulation strategies, reducing morbidity and mortality in AF patients.

## 1. Introduction

Atrial fibrillation (AF) is the most common cardiac arrhythmia, affecting approximately 1% of individuals under 60 years and over 12% of those aged 75–84. It accounts for one-third of hospitalizations due to cardiac rhythm disorders, posing a significant public health burden. AF increases the risk of stroke by fivefold, heart failure by threefold, and dementia and mortality by twofold [1]. Effective management strategies, including rate control, rhythm control, and thromboembolism prevention, rely on oral anticoagulants (OACs) such as warfarin or non-vitamin K antagonist oral anticoagulants (NOACs) like dabigatran, rivaroxaban, apixaban, and edoxaban [2,3]. While OACs significantly reduce thromboembolic events, they elevate the risk of gastrointestinal (GI) bleeding, a leading cause of morbidity and mortality in AF patients [1,2,4].

The challenge of balancing thromboembolism prevention with bleeding risk is compounded by overlapping risk factors, such as advanced age, hypertension, and renal dysfunction [5]. The CHA_2_DS_2_-VASc score is widely used to assess stroke risk, guiding OAC initiation, while the HAS-BLED score predicts bleeding risk, as recommended by the 2014 AHA/ACC/HRS guidelines [6]. However, these scores have limitations in patients with complex profiles, including polypharmacy or multiple comorbidities, where predictive accuracy may be suboptimal [7].

Recent studies have identified systemic inflammatory markers, such as the neutrophil-to-lymphocyte ratio (NLR), platelet-to-lymphocyte ratio (PLR), systemic immune-inflammation index (SII), and uric acid/albumin ratio, as predictors of adverse outcomes in cardiovascular and inflammatory diseases [8,9,10,11]. These biomarkers, derived from routine blood tests, are cost-effective and readily available in the ED, making them practical for rapid risk assessment [12]. The NLR is associated with poor prognosis in GI bleeding and incorporating it into established risk scores, such as the Glasgow-Blatchford and Rockall scores, may enhance their predictive power [13]. Similarly, PLR and SII have shown prognostic value in conditions such as liver cirrhosis and intracranial hemorrhage [14,15]. The uric acid/albumin ratio, which reflects oxidative stress and inflammation, predicts mortality in elderly GI bleeding patients [16].

Additionally, low cholesterol and albumin levels are associated with systemic inflammation, malnutrition, and liver dysfunction, and are thought to increase the risk of bleeding by disrupting haemostatic mechanisms.

This study aims to evaluate the predictive value of these inflammatory markers for GI bleeding in AF patients receiving OACs, comparing NOAC- and warfarin-treated individuals to identify differences in biomarker profiles. We hypothesize that using inflammatory indices, combined with biochemical parameters like hypoalbuminemia and hypolipidemia (total cholesterol < 120 mg/dL or low-density lipoprotein cholesterol (LDL) < 50 mg/dL), will enhance risk stratification beyond traditional scores.

## 2. Materials and Methods

### 2.1. Study Design and Setting

This retrospective cohort study was conducted in the Emergency Department of Balikesir University Hospital between 2019 and 2023. The primary objective was to assess the association between systemic inflammatory markers and GI bleeding in AF patients receiving OACs, focusing on their utility in the ED.

### 2.2. Study Population

Adult patients (≥18 years) with electrocardiography-confirmed AF, prescribed OACs (warfarin or NOACs), and presenting to the ED were included. Patients were divided into

- Case Group: AF patients receiving OACs with confirmed GI bleeding (hematemeza, melena, or hematochezia), verified by endoscopy or imaging where applicable.
- Control Group: AF patients receiving OACs presenting for non-bleeding reasons (e.g., routine follow-up, unrelated complaints) during the same period.

Exclusion criteria included

- Incomplete medical records or missing laboratory data.
- Anticoagulation for non-AF indications (e.g., venous thromboembolism, mechanical valves).
- Non-GI bleeding (e.g., epistaxis, hematuria).
- Active malignancy, acute infection, or conditions confounding inflammatory marker levels.

### 2.3. Data Collection

The following data were extracted from electronic medical records:

- Demographic Characteristics: Age, sex.
- Clinical Data: Comorbidities (hypertension, diabetes, heart failure, prior bleeding, renal or liver dysfunction), medication use (OAC type, proton pump inhibitors [PPIs], antiplatelets), and endoscopic findings.
- Risk Scores: CHA_2_DS_2_-VASc (stroke risk) and HAS-BLED (bleeding risk) scores.
- Laboratory Parameters: Hemoglobin, urea, creatinine, glomerular filtration rate (GFR), albumin, uric acid, C-reactive protein (CRP), total cholesterol, low-density lipoprotein (LDL), high-density lipoprotein (HDL), triglycerides, and complete blood count (CBC) with differential (neutrophils, lymphocytes, platelets).
- Inflammatory Indices:
  ○ Uric acid/albumin ratio = uric acid (mg/dL)/albumin (g/L).
  ○ CRP/albumin ratio = CRP (mg/L)/albumin (g/L).
  ○ Neutrophil-to-lymphocyte ratio (NLR) = neutrophil count/lymphocyte count.
  ○ Platelet-to-lymphocyte ratio (PLR) = platelet count/lymphocyte count.
  ○ Systemic immune-inflammation index (SII) = (platelet count × neutrophil count)/lymphocyte count.
  ○ Triglyceride–glucose (TyG) index = ln [triglyceride (mg/dL) × fasting glucose (mg/dL)/2].
  ○ Atherogenic index of plasma (AIP) = log (triglyceride/HDL cholesterol).

Laboratory samples were collected at ED admission and processed using standardized analyzers (e.g., Beckman Coulter (Brea, CA, USA) for CBC, Roche Cobas (Basel, Switzerland) for biochemistry). INR was measured in warfarin users to assess anticoagulation intensity.

### 2.4. Statistical Analysis

Data were analyzed using SPSS version 20.0 (IBM Inc., Chicago, IL, USA). Normality was assessed via the Shapiro–Wilk test. Continuous variables were reported as mean ± standard deviation (SD) for normal distributions or median (minimum–maximum) for non-normal distributions. Categorical variables were expressed as frequencies and percentages. The comparisons used were as follows:

- Independent *t*-test for normally distributed continuous variables.
- Mann–Whitney U test for non-normally distributed continuous variables.
- Chi-squared test or Fisher’s exact test for categorical variables.

Subgroup analyses explored differences by OAC type, age (<75 vs. ≥75 years), HAS-BLED score (≤2 vs. >2), and NOAC type (where available). Odds ratios (ORs) with 95% confidence intervals (CIs) were calculated for key predictors. A *p*-value <0.05 was considered significant. Multivariate regression was not performed due to the retrospective design and sample size limitations.

## 3. Results

### 3.1. Demographic and Clinical Characteristics

The study included 155 patients: 78 in the NOAC group (42 cases, 36 controls) and 77 in the warfarin group (43 cases, 34 controls). Demographic and clinical characteristics are presented in Table 1.

Age, sex, and most comorbidities were comparable between cases and controls. Prior bleeding history was significantly higher in NOAC cases (*p* = 0.048; OR: 3.21, 95% CI: 1.01–10.23) and trended higher in warfarin cases (*p* = 0.064; OR: 2.78, 95% CI: 0.91–8.47). PPI use was similar, suggesting consistent gastroprotective strategies [4]. HAS-BLED scores were significantly higher in bleeding groups (*p* < 0.001), with means of 3.5 (NO-ACs) and 3.7 (warfarin) in cases versus 2.6 and 2.8 in controls. CHA_2_DS_2_-VASc scores showed no significant differences, indicating similar stroke risk profiles [5].

Subgroup analysis by age (<75 vs. ≥75 years) revealed that older NOAC users (≥75) had a higher prevalence of prior bleeding (*p* = 0.042; OR: 2.14, 95% CI: 1.03–4.45) and heart failure (*p* = 0.039; OR: 2.00, 95% CI: 1.04–3.85). In warfarin users, older patients had higher HAS-BLED scores (4.0 ± 1.2 vs. 3.3 ± 1.1, *p* = 0.011). Sex-based analysis showed no significant differences, though males in the warfarin group had a slightly higher hypertension prevalence (75.0% vs. 68.0%, *p* = 0.091). Analysis by NOAC type (available for 70% of NOAC cases: 20 rivaroxaban, 15 apixaban, 7 dabigatran) showed that rivaroxaban users had a higher bleeding prevalence (55.0% vs. 40.0% for apixaban, *p* = 0.082), though sample size limited statistical power.

### 3.2. Laboratory Parameters

Laboratory findings are detailed in Table 2.

In NOAC users, cases had lower hemoglobin (*p* < 0.001; OR: 0.65, 95% CI: 0.52–0.81), albumin (*p* < 0.001; OR: 0.88, 95% CI: 0.82–0.94), and lipid levels (total cholesterol *p* < 0.001; LDL *p* = 0.002; HDL *p* = 0.003), with higher uric acid (*p* = 0.024; OR: 1.32, 95% CI: 1.04–1.68) and GFR (*p* = 0.042). Creatinine was lower in cases (*p* = 0.032). In warfarin users, cases had lower hemoglobin (*p* < 0.001; OR: 0.63, 95% CI: 0.50–0.79), albumin (*p* < 0.001; OR: 0.87, 95% CI: 0.81–0.93), and lipid levels (total cholesterol *p* = 0.021; LDL *p* = 0.041), with higher urea (*p* = 0.012; OR: 1.18, 95% CI: 1.03–1.35) and INR (*p* < 0.001; OR: 3.12, 95% CI: 1.98–4.91).

Subgroup analysis by HAS-BLED score (>2 vs. ≤2) showed that NOAC users with high scores had lower hemoglobin (*p* = 0.008) and albumin (*p* = 0.003). In warfarin users, high HAS-BLED scores correlated with higher INR (*p* < 0.001) and urea (*p* = 0.019). Age-based analysis indicated that older NOAC users (≥75) had lower albumin (*p* = 0.021) and higher uric acid (*p* = 0.033). In rivaroxaban users, albumin was lower in cases (*p* = 0.002), while apixaban users showed no significant differences.

### 3.3. Inflammatory Indices

Inflammatory indices are shown in Table 3.

In NOAC users, cases had significantly higher uric acid/albumin ratio, PLR (*p* = 0.037; OR: 1.01, 95% CI: 1.00–1.02), NLR (*p* = 0.009; OR: 1.15, 95% CI: 1.04–1.28), and SII (*p* = 0.005; OR: 1.00, 95% CI: 1.00–1.01) values. In warfarin users, only the uric acid/albumin ratio was significantly elevated (*p* < 0.001; OR: 2.45, 95% CI: 1.43–4.20). Subgroup analysis showed that older NOAC users (≥75) had higher NLR (*p* = 0.014) and SII (*p* = 0.008). High HAS-BLED scores (>2) correlated with higher SII in NOAC cases (*p* = 0.012). In rivaroxaban users, SII was higher in cases (*p* = 0.010).

### 3.4. Endoscopic Findings

Endoscopic data were available for 80% of cases (34/42 NOAC, 35/43 warfarin). In NOAC users, common findings included gastric ulcers (35.3%), duodenal ulcers (23.5%), and erosive gastritis (20.6%). In warfarin users, gastric ulcers (37.1%), duodenal ulcers (20.0%), and angiodysplasia (14.3%) were prevalent. Forrest classification showed a higher proportion of high-risk lesions ((active spurter, active oozing, non-bleeding visible vessel, adherent clot) Forrest Ia–IIb) in NOAC users (47.1% vs. 37.1% in warfarin, *p* = 0.089). Elevated urea in warfarin cases correlated with high-risk lesions (*p* = 0.027) [17]. Rivaroxaban users had a higher prevalence of high-risk lesions (50.0% vs. 40.0% for apixaban, *p* = 0.101)

## 4. Discussion

This study provides comprehensive evidence that systemic inflammatory markers and biochemical parameters predict GI bleeding in AF patients taking OACs, with distinct profiles for patients receiving NOAC or warfarin. In individuals receiving NOACs, elevated uric acid/albumin ratio, NLR, PLR, and SII scores, hypoalbuminemia, and hypolipidemia were significantly associated with bleeding risk [8,9,10,11]. In individuals receiving warfarin, the uric acid/albumin ratio, hypoalbuminemia, elevated urea, and supratherapeutic INR were key predictors [16,17,18]. The HAS-BLED score effectively identified high-risk patients, but integrating inflammatory indices could enhance its predictive accuracy, particularly for patients receiving NOACs [6]. These findings high-light the potential of routine ED blood tests to guide risk stratification and clinical management.

### 4.1. Inflammatory Markers: Mechanisms and Implications

Systemic inflammation is a critical driver of GI bleeding in anticoagulated patients, as neutrophils, lymphocytes, and platelets interact in inflammatory and hemostatic pathways [8]. NLR, reflecting neutrophil-driven inflammation, was significantly elevated in NOAC cases. Neutrophils promote mucosal injury through the release of reactive oxygen species and cytokines, increasing bleeding propensity [8]. Chen et al. reported that using NLRs is associated with better prognostic scores (Glasgow-Blatchford and Rockall) for upper GI bleeding, supporting its utility in the ED [13]. The lack of statistical significance for NLR in patients receiving warfarin (*p* = 0.364) may reflect this drug’s primary effect on coagulation rather than inflammation [19]. This difference suggests that NOACs may amplify inflammatory responses, possibly via protease-activated receptor (PAR) pathways, as noted by Ingrasciotta et al. [19].

PLR, indicative of platelet activation and inflammatory burden, was elevated in NOAC-treated cases. Wang et al. found similar associations in variceal bleeding, suggesting that PLR reflects inflammatory stress and mucosal injury [14]. Platelets contribute to hemostasis but also release pro-inflammatory mediators, exacerbating bleeding in inflamed tissues [8]. SII, integrating neutrophil, lymphocyte, and platelet counts, was significantly higher in NOAC-treated cases. Trifan et al. and Liang et al. linked SII to poor outcomes in intracerebral hemorrhage, supporting its prognostic value in bleeding events [15,20]. The higher SII in patients receiving rivaroxaban (*p* = 0.010) suggests drug-specific inflammatory effects that warrant further investigation.

The uric acid/albumin ratio was a robust predictor in both groups. Uric acid promotes endothelial dysfunction and oxidative stress, increasing vascular permeability and bleeding risk [21]. Hypoalbuminemia, a negative acute-phase reactant, amplifies the sensitivity of this ratio [16]. The consistency across different types of OAC positions this ratio as a practical biomarker for ED risk assessment.

### 4.2. Biochemical Predictors: Clinical and Pathophysiological Insights

Hypoalbuminemia was a consistent predictor across both groups. Low albumin levels reflect inflammation, malnutrition, or liver dysfunction, which are all associated with increased bleeding risk [21,22]. In our study, consistent with the literature, patients receiving rivaroxaban had lower albumin levels, suggesting that the drug has specific effects on protein binding (*p* = 0.002) [21,22,23].

Hypolipidemia emerged as a novel predictor in patients receiving NOAC. Low cholesterol levels are associated with systemic inflammation, malnutrition, and liver dysfunction, impairing hemostatic mechanisms [24,25]. In patients receiving warfarin, lipid differences were less pronounced, likely due to warfarin’s primary coagulation effects.

The elevated levels of urea in patients receiving warfarin correlated with high-risk endoscopic lesions, consistent with Chopra et al.’s findings that urea reflects intestinal protein breakdown during bleeding [17]. Supratherapeutic INR in warfarin cases underscores the importance of therapeutic monitoring, as noted by Vinogradova et al. [18]. Elevated uric acid in patients receiving NOAC may contribute to endothelial dysfunction [21].

### 4.3. Renal Function and Bleeding Risk

The higher GFRs and lower creatinine levels in NOAC-treated cases contrast with reports linking renal impairment to bleeding risk [18,26]. Vinogradova et al. noted that NOACs increase bleeding risk in severe renal failure, but our cohort’s preserved renal function suggests effective dose optimization [18]. Yao et al. emphasized the importance of dose adjustments in renal dysfunction, which may explain our findings [26]. These results highlight the need for renal function monitoring in NOAC therapy, particularly in elderly patients.

### 4.4. Clinical Scores: Strengths and Limitations

The HAS-BLED score effectively identified high-risk patients (*p* < 0.001), aligning with Gallego et al.’s validation [6]. Subgroup analysis showed that high HAS-BLED scores (>2) correlated with lower hemoglobin, lower albumin, and higher SII in NOAC cases, reinforcing its utility when combined with biomarkers [7]. CHA_2_DS_2_-VASc scores showed no differences, reflecting their specificity for stroke risk [5]. Integrating these markers into risk scores could improve predictive accuracy in the ED.

### 4.5. Emergency Department Management Strategies

The management of AF patient GI bleeding in the ED requires rapid risk stratification to guide interventions, such as endoscopy, PPI therapy, and anticoagulation reversal [1,4]. Elevated NLR, PLR, SII, and uric acid/albumin ratio in patients receiving NOAC could prompt earlier endoscopic intervention or closer monitoring. In patients receiving warfarin, elevated INR and urea necessitate urgent INR correction (e.g., vitamin K, fresh frozen plasma) and endoscopic evaluation [4].

Endoscopic findings revealed a higher prevalence of high-risk lesions (Forrest Ia–IIb) in patients receiving NOAC, suggesting that inflammatory markers may correlate with lesion severity [17]. PPI use was similar across groups, but its protective effect may be limited in patients receiving anticoagulants, as noted by Ballestri et al. [4]. In patients receiving rivaroxaban, high-risk lesions were more prevalent, aligning with Vinogradova et al.’s findings of higher bleeding risk with rivaroxaban treatment [18]. Anticoagulation reversal strategies (e.g., idarucizumab for dabigatran, andexanet alfa for factor Xa inhibitors) should be considered in severe cases, as recommended by Martin et al. [1].

### 4.6. Subgroup Analyses and Personalized Medicine

Subgroup analyses provided nuanced insights. Older NOAC users (≥75) had higher NLR and SII values, indicating age-related inflammation as a risk factor. High HAS-BLED scores (>2) correlated with more severe biomarker abnormalities, supporting biomarker integration into risk scores [7]. Patients receiving rivaroxaban exhibited higher SII values and lower albumin levels, suggesting drug-specific effects [19]. These findings align with Connolly et al.’s RE-LY trial, which reported comparable bleeding risks between dabigatran and warfarin; however, our study suggests NOACs may have distinct inflammatory profiles [2].

Personalized anticoagulation strategies are critical to balance bleeding and thromboembolic risks [2]. Patients with elevated inflammatory markers or hypoalbuminemia may benefit from lower-dose NOACs or alternative agents. In patients receiving warfarin, strict INR monitoring is essential, as supratherapeutic levels significantly increase bleeding risk [18]. Adjunctive therapies, such as anti-inflammatory agents or nutritional support, could mitigate bleeding risk in high-risk patients, though further research is needed.

### 4.7. Limitations and Future Directions

The retrospective design and moderate sample size (n = 155) of this study limit the generalizability of the findings. Selection bias may have occurred, as controls had higher renal and liver dysfunction rates, possibly due to stricter monitoring [26]. The study did not fully differentiate NOAC types due to incomplete data, though rivaroxaban showed trends toward a higher bleeding risk [18]. Endoscopic data were incomplete for 20% of cases, potentially underestimating lesion severity. The lack of multivariate analysis limits confounder adjustment.

Future research should include

- Prospective, multicenter studies to validate biomarkers in larger cohorts.
- Analysis of specific NOAC types and dosages to identify drug-specific risks.
- Integration of inflammatory markers into established risk scores such as the HAS-BLED or Glasgow-Blatchford scores.
- Exploration of anti-inflammatory therapies to reduce bleeding risk.
- Investigation of hypolipidemia’s role in bleeding risk, particularly in patients receiving NOAC.

## 5. Conclusions

This study demonstrates that systemic inflammatory markers (uric acid/albumin ratio, NLR, PLR, SII) and biochemical parameters (hypoalbuminemia, hypolipidemia, elevated urea, INR) are valuable predictors of GI bleeding in AF patients receiving OACs. Patients receiving NOACs exhibit a distinct inflammatory profile, with elevated NLR, PLR, and SII values, while patients receiving warfarin show significant changes in uric acid/albumin ratio, urea levels, and INR. The HAS-BLED score remains a robust tool, but integrating inflammatory indices could enhance its accuracy, particularly for patients receiving NOAC. Routine ED blood tests offer a cost-effective approach to risk stratification, guiding interventions like endoscopy, PPI therapy, and anticoagulation reversal. Personalized strategies, tailored to biomarker profiles, age, and comorbidities, could reduce morbidity and mortality. Prospective studies are needed to validate these biomarkers and integrate them into clinical algorithms, which will improve outcomes in AF patients receiving OACs.

## Figures and Tables

**Table 1:** Demographic and Clinical Characteristics of NOAC and Warfarin Users.

Parameter	NOAC Users			Warfarin Users		
	Case Group (n = 42)	Control Group (n = 36)	*p*-Value	Case Group (n = 43)	Control Group (n = 34)	*p*-Value
Age (years) *	74.2 ± 8.7	71.8 ± 9.1	0.245	73.9 ± 9.0	72.1 ± 8.8	0.389
Male, n (%)	20 (47.6)	18 (50.0)	0.832	22 (51.2)	17 (50.0)	0.913
Hypertension, n (%)	30 (71.4)	25 (69.4)	0.842	31 (72.1)	23 (67.6)	0.662
Diabetes, n (%)	15 (35.7)	12 (33.3)	0.818	16 (37.2)	11 (32.4)	0.652
Heart Failure, n (%)	18 (42.9)	10 (27.8)	0.159	20 (46.5)	12 (35.3)	0.315
Prior Bleeding, n (%)	12 (28.6)	4 (11.1)	0.048	14 (32.6)	5 (14.7)	0.064
Renal Dysfunction, n (%)	8 (19.0)	12 (33.3)	0.139	9 (20.9)	11 (32.4)	0.234
Liver Dysfunction, n (%)	3 (7.1)	6 (16.7)	0.185	4 (9.3)	7 (20.6)	0.149
PPI Use, n (%)	25 (59.5)	20 (55.6)	0.711	26 (60.5)	19 (55.9)	0.678
CHA_2_DS_2_-VASc *	4.1 ± 1.6	3.8 ± 1.5	0.376	4.0 ± 1.7	3.7 ± 1.6	0.412
HAS-BLED *	3.5 ± 1.2	2.6 ± 1.0	<0.001	3.7 ± 1.3	2.8 ± 1.1	<0.001

* Mean ± SD.

**Table 2:** Laboratory Parameters of NOAC and Warfarin Users.

Parameter	NOAC Users			Warfarin Users		
	Case Group (n = 42)	Control Group (n = 36)	*p*-Value	Case Group (n = 43)	Control Group (n = 34)	*p*-Value
Hemoglobin (g/dL) *	9.8 ± 2.1	12.5 ± 1.9	<0.001	9.5 ± 2.0	12.3 ± 1.8	<0.001
Urea (mmol/L) **	8.2 (4.1–15.6)	7.5 (3.8–14.2)	0.214	9.1 (4.5–16.8)	7.3 (3.9–13.5)	0.012
Creatinine (µmol/L) **	85.0 (50–130)	95.0 (60–150)	0.032	90.0 (55–140)	92.0 (60–145)	0.456
GFR (mL/min/1.73 m^2^) *	65.2 ± 15.3	58.7 ± 14.8	0.042	62.4 ± 14.7	60.8 ± 15.1	0.627
Albumin (g/L) *	32.1 ± 5.4	38.2 ± 4.9	<0.001	31.8 ± 5.2	37.5 ± 4.7	<0.001
Uric Acid (mg/dL) *	6.8 ± 1.7	5.9 ± 1.5	0.024	6.5 ± 1.6	6.0 ± 1.4	0.134
CRP (mg/L) **	12.5 (2.0–150.0)	10.0 (1.5–100.0)	0.126	13.0 (2.5–140.0)	11.0 (1.8–90.0)	0.154
Total Cholesterol (mg/dL) *	145.3 ± 30.2	170.5 ± 28.7	<0.001	150.2 ± 29.8	165.7 ± 27.5	0.021
LDL (mg/dL) *	85.6 ± 22.4	100.4 ± 20.8	0.002	88.4 ± 21.6	98.2 ± 19.7	0.041
HDL (mg/dL) *	38.2 ± 10.1	45.7 ± 9.8	0.003	40.1 ± 9.9	43.8 ± 10.2	0.112
Triglycerides (mg/dL) **	110.0 (60–200)	120.0 (70–220)	0.091	115.0 (65–210)	125.0 (75–230)	0.102
INR *	-	-	-	3.8 ± 1.2	2.4 ± 0.8	<0.001

* Mean ± SD, ** Median (min–max).

**Table 3:** Inflammatory Indices in NOAC and Warfarin Users.

Parameter	NOAC Users			Warfarin Users		
	Case Group (n = 42)	Control Group (n = 36)	*p*-Value	Case Group (n = 43)	Control Group (n = 34)	*p*-Value
Uric Acid/Albumin *	2.2 ± 0.8	1.6 ± 0.5	<0.001	2.0 ± 0.6	1.6 ± 0.4	<0.001
CRP/Albumin **	1.7 (0.7–54.6)	1.2 (0.6–29.1)	0.082	1.8 (0.7–48.4)	1.3 (0.7–18.1)	0.111
PLR **	171.4 (54.0–1476.7)	134.0 (29.7–833.3)	0.037	172.7 (76.4–970.0)	145.9 (55.7–375.5)	0.134
NLR **	4.6 (1.0–29.3)	2.5 (1.0–28.7)	0.009	3.6 (1.1–37.0)	2.9 (0.7–11.2)	0.364
SII **	872.5 (162.5–12994.7)	586.5 (256.6–7166.7)	0.005	804.1 (280.3–17945.0)	616.9 (43.0–3039.1)	0.105
TyG Index *	3.8 ± 0.3	3.8 ± 0.3	0.677	3.9 ± 0.2	3.8 ± 0.3	0.573
AIP *	0.4 ± 0.2	0.4 ± 0.2	0.806	0.4 ± 0.3	0.4 ± 0.2	0.772

* Mean ± SD, ** Median (min–max).

## Data Availability

Due to privacy concerns, the data presented in this study are available from the corresponding author upon request.
